# Reflection Spectroscopy Skin Carotenoids Correlate with Serum Carotenoids in School-Aged Children

**DOI:** 10.1016/j.tjnut.2026.101406

**Published:** 2026-02-11

**Authors:** Ruyu Liu, Laura M Rosok, Molly Black, John W Erdman, Naiman A Khan

**Affiliations:** 1 Department of Health and Kinesiology, University of Illinois, Urbana, IL, United States; 2 Department of Neurology, Washington University, St. Louis, MO, United States; 3 Department of Food Science and Human Nutrition, University of Illinois, Urbana, IL, United States

**Keywords:** fruit and vegetable intake, veggie meter, adolescents, pediatric, dietary assessment

## Abstract

**Background:**

Serum carotenoids are thought to reflect higher consumption of fruits and vegetables. However, venous blood draws are invasive and not suitable for pediatric populations. Reflection spectroscopy (RS) offers a noninvasive alternative for measuring carotenoids in the skin; however, the relationship between RS-assessed skin carotenoids and serum carotenoids in children remains understudied.

**Objectives:**

This study aimed to test the hypothesis that RS-assessed skin carotenoids would significantly correlate with serum and dietary carotenoids in children.

**Methods:**

This was a cross-sectional study among children (*n* = 51, 11.0 ± 1.9 y). Serum carotenoids were quantified using high-performance liquid chromatography. Skin carotenoids were assessed using the Veggie Meter, a pressure-mediated RS device. Dietary carotenoids were measured via 7-d food diaries and analyzed using the Nutrition Data Systems for Research software. Height and weight were measured to calculate the BMI percentile. Log transformation was applied to all carotenoid variables to address right-skewness. Pearson’s partial correlations were conducted, adjusting for age, sex, and BMI percentile. Benjamini–Hochberg false discovery rate (FDR) correction was applied for multiple comparisons.

**Results:**

Most participants (76%) had a normal BMI percentile for age and sex. Skin carotenoids significantly correlated with serum lycopene (*r =* 0.31, *P =* 0.034), β-cryptoxanthin (*r =* 0.49, *P* < 0.001), β-carotene (*r =* 0.75, *P* < 0.001), lutein (*r =* 0.45, *P =* 0.002), zeaxanthin (*r =* 0.36, *P =* 0.013), and total carotenoids (*r =* 0.65, *P* < 0.001). Skin carotenoids were significantly correlated with several dietary carotenoids, including β-carotene (*r =* 0.48, *P =* 0.0095), α-carotene (*r =* 0.38, *P =* 0.041), lutein/zeaxanthin (*r =* 0.37, *P* = 0.041), and dark green vegetable intake (*r =* 0.43, *P =* 0.033). Dietary and serum carotenoids were not significantly correlated with each other after FDR correction.

**Conclusions:**

RS-assessed skin carotenoids demonstrated robust correlations with serum carotenoids, supporting their utility as a noninvasive biomarker of carotenoid status in school-aged children.

## Introduction

Children and adolescents fall short of the recommended intake of fruits and vegetables (FV) in the United States and globally [1,2]. Evidence from the United States Center for Disease Control and Prevention showed that one in 2 children aged 1–5 y did not consume a daily vegetable during the preceding week [3]. Only 7.1% and 2.0% of adolescents met the intake recommendations for FV, respectively [4]. Notably, the gap between actual intake and recommended intake widens as children transition into young adulthood [2]. This is concerning, given that FV-rich diets provide important nutrients and bioactives for optimal growth and are associated with reduced risks for hypertension, type 2 diabetes, and some cancers in adulthood [5,6]. Moreover, childhood represents a critical window for establishing healthy dietary patterns, as eating behaviors developed in early life tend to persist into adulthood [7]. These factors underscore the urgent need for effective interventions to increase FV consumption in pediatric populations. Such interventions, however, depend on accurate dietary assessment methods. Although recall-based, self-report approaches [e.g., food frequency questionnaires (FFQ), 24-h diet recalls, and diet records) are widely used due to their convenience and affordability, children have difficulty with portion size estimation and are prone to both underreporting and over-reporting their intake [8]. Parental reporting introduces additional sources of error, including limited knowledge of foods consumed away from home and social desirability bias. Furthermore, parents' own dietary patterns and weight status may influence the accuracy of their reporting [8]. Therefore, reliable biomarkers that reflect dietary FV intake in children are needed.

The blood concentrations of carotenoids have been identified as the target biomarker for FV intake [9]. Carotenoids are fat-soluble nutrients abundantly found in FV, particularly yellow, orange, red, and dark green vegetables. Humans cannot produce carotenoids endogenously and must obtain them through diet. Once ingested, carotenoids circulate in the bloodstream and deposit in various tissues such as the adipose tissue, liver, adrenals, skin, macula, and brain [10]. The most prevalent dietary carotenoids in the United States include β- and α-carotene, lycopene, lutein, zeaxanthin, and β-cryptoxanthin [9]. The reference standard for assessing blood concentrations of carotenoids is plasma or serum carotenoids through blood draws using HPLC. However, this technique is invasive and costly, and obtaining blood draws from children can be particularly difficult, especially in a field-based setting. Given these constraints, skin carotenoids have recently emerged as a surrogate measure.

The most widely used skin carotenoid assessment tools are resonance Raman spectroscopy (RRS) and reflection spectroscopy (RS) [[11], [12], [13], [14], [15]]. RRS uses 480 and 514.5 nm laser excitation to resonantly enhance Raman scattering from the conjugated carbon backbone common to all carotenoid molecules, measuring the combined concentration of β-carotene, lycopene, β-cryptoxanthin, lutein, zeaxanthin, and their isomers [[11], [12], [13]]. Although the high-power light source in RRS enables high molecule-specificity, it also makes the instrument more costly, bulky, and sensitive to vibrations, thus undesirable to transport [14,15]. RS is a newer method to measure skin carotenoids. Similar to RRS, RS quantifies carotenoids by measuring their light absorption strength. However, RS uses broadband light sources, making the instrument comparatively less expensive and more portable. The RS scores indicate the absorption strength of chromophores in the 460- to 520-nm wavelength range, encompassing α- and β-carotenes, β-cryptoxanthin, lycopene, lutein, and zeaxanthin, while minimizing the potential interference effect induced by oxy- and deoxyhemoglobin and melanin [16]. A 2020 systematic review demonstrated moderate to strong correlations between spectroscopy-assessed skin carotenoids and dietary intake (*r =* 0.38–0.69) and blood carotenoids (*r =* 0.62–0.78). However, only 4 of the 29 included studies were conducted among school-aged children (i.e., aged 6 y and above), none of which used RS to assess skin carotenoids [17]. Since the review was published, evidence has shown that RS-assessed skin carotenoids moderately to strongly correlated with serum or plasma carotenoids in adults (*r =* 0.52–0.72) [[18], [19], [20]]. However, the correlations between RS-assessed skin carotenoids and blood concentrations of carotenoids in children have been understudied. One study conducted in Nepal found moderate and significant correlation (*r =* 0.53) between total serum carotenoids and RS-assessed skin carotenoids in a child sample (aged 8–12 y). However, serum carotenoids were not individually examined [21]. This knowledge gap limits the broader application of RS as an FV biomarker, as correlations in adults cannot be directly extrapolated to children due to physiological and dietary differences [2,22,23]. Evidence filling this gap may support the utility of RS in behavior change intervention and surveillance efforts in school settings.

To address this knowledge gap, the primary objective of this study was to determine the correlations between RS-assessed skin carotenoids and serum carotenoids in a child sample. The secondary objectives included examining correlations between dietary carotenoid intake and both skin and serum carotenoids, as well as assessing differences in RS scores across demographic and anthropometric characteristics.

## Methods

### Study design

This work was a secondary analysis of a cross-sectional study examining metabolic health and cognitive function in children. Inclusion criteria included the following: *1*) aged between 7 and 13 y, *2*) no prior diagnosis of neurologic disorders, metabolic disease (e.g., diabetes, cardiovascular disease), or anemia over the preceding year, and *3*) no blood donation within 8 wk before study enrollment. Participants were recruited through flyers distributed in community centers, schools, and summer camps in the Champaign-Urbana area, as well as University newsletter emails and advertisement on Facebook. The study was approved by the University of Illinois Urbana-Champaign Institutional Review Board (IRB: 19811).

### Outcome measures

#### Dietary carotenoids

The parent or guardian of the child completed a 7-d food diary to record habitual dietary intake of the child participant [24]. Graduate research assistants who were trained by a registered dietitian provided the parents or guardians with instructions on how to complete the record. Briefly, the record included the time and location the food/beverage was consumed, the type of food/beverage (including brand names whenever possible) consumed, detailed descriptions of the food/beverage (including how the food was prepared and all ingredients added while cooking), and the amount consumed in measurable terms such as cups, ounces, and teaspoons. Parents and guardians were also instructed to record any dietary supplements, including brand, type, quantity, and units. A portion size guide (e.g., 3 oz cooked meat = deck of cards) and a prefilled sample record were provided in the food diary booklet. The food diary data were entered and analyzed using Nutrition Data Systems for Research software version 2022, developed by the Nutrition Coordinating Center, University of Minneapolis. Total fruit intake was the sum of daily servings consumed of citrus juice, fruit juice excluding citrus juice, citrus fruit, fruit excluding citrus fruit, avocado, and similar, and fried fruits. Total vegetable intake was the sum of daily servings consumed of dark green vegetables, deep yellow vegetables, tomato, white potatoes, fried potatoes, other starchy vegetables, legumes and beans, other vegetables, fried vegetables, and vegetable juice. Total fruit and vegetable intake was the sum of total fruit intake and total vegetable intake.

#### Serum carotenoids

Venous blood draws were conducted after ≥4-h fast. Before the blood draw, the site was cleansed with 70% isopropyl alcohol pads. The blood was collected into evacuated tubes (Vacutainer; Becton Dickinson) coated with ethylene diaminetetraacetic acid as an anticoagulant. Serum was also collected into silicone-coated tubes (Vacutainer; Becton Dickinson). Serum carotenoid concentrations were determined using HPLC with photodiode array detection, as previously described [25]. Briefly, serum samples (300 μL) were mixed with an equal volume of ethanol containing 0.1% butylated hydroxytoluene and vortexed. Carotenoids were extracted using 3 consecutive hexane extractions, with organic layers combined and dried under nitrogen. Extracts were reconstituted in 90% methyl tert-butyl ether, 8% methanol, and 2% aqueous ammonium acetate solution and analyzed using a Waters Alliance HPLC system (e2695 Separation Module) equipped with a 2998 photodiode array detector and YMC reverse-phase C30 column (4.6 × 150 mm, 3 μm). Target carotenoids, including lycopene, lutein, zeaxanthin, β-carotene, and β-cryptoxanthin, were quantified using calibration curves prepared from authenticated standards (Carotenature) with compound-specific extinction coefficients. All procedures were performed under subdued lighting to prevent photodegradation of light-sensitive carotenoids. Internal standards were not used in the analysis; however, analytical precision was demonstrated through blinded split samples with acceptable coefficients of variation (6%–11%) for all target carotenoids [26].

#### Skin carotenoids

Skin carotenoids were assessed noninvasively using the Veggie Meter (Longevity Link Corporation) which is a pressure-mediated RS device [14]. The ring finger of the nondominant hand was cleaned with 70% isopropyl alcohol pad and used for the scans. The finger was removed and repositioned into the device after each scan. The Veggie Meter scores are logarithmic scale values based on reflectivity, ranging from 0 to 800, with higher scores indicating higher skin carotenoids [16]. The mean scores of 3 measurements were used in the analyses.

#### Anthropometrics

Height and weight were assessed without shoes using a stadiometer (Seca, model 240) and a Tanita WB-300 Plus digital scale, respectively. The average values of 3 measurements were used for the analyses. BMI percentile was calculated based on the United States Centers for Disease Control and Prevention growth charts accounting for age and sex [27].

#### Demographic characteristics

Information on child age, sex, race, ethnicity, parental education concentrations, and household annual income was collected using an online survey completed by parent or guardian of the child participant.

#### Statistical analysis

All data analyses were conducted in RStudio (Version 2025.05.1 + 513). Statistical significance was defined as *P <* 0.05. Descriptive statistics (i.e., counts, proportions, means, and SDs) were utilized to summarize sample characteristics. To assess the reproducibility of skin carotenoid measurements, the coefficient of variation was calculated as the SD divided by the mean, expressed as a percentage, to measure the precision of repeated 3 measurements within each participant. The intraclass correlation coefficient was calculated using a 2-way random effects model to assess the consistency of measurements. Normality of all carotenoid variables, total energy intake, and macronutrient variables was assessed using the Shapiro–Wilk test, and skewness was assessed through visual inspection of histograms for nonnormal variables. All carotenoid variables and protein intake were right-skewed. Total energy, total carbohydrate, and total fat intake were normally distributed (Shapiro–Wilk *P* > 0.05). Therefore, Mann–Whitney U, Kruskal–Wallis, and Spearman correlations were used to detect differences in skin, serum, and dietary intake by demographic and anthropometric characteristics. The following 4 sets of Pearson’s partial correlations were conducted to determine the correlations between: *1*) skin carotenoids and individual and total serum carotenoids, *2*) skin carotenoids and individual and total dietary carotenoids, *3*) skin carotenoids and food group intakes, and *4*) individual and total dietary and serum carotenoids. All partial correlations were adjusted for a priori covariates, including age, sex, and BMI percentile, based on previous literature showing their effect on carotenoid status [28]. All carotenoid variables were log-transformed to address right-skewness. The normality of each log-transformed variable was visually examined and found to be acceptable. The outliers remaining after log transformation (*n* = 6 among 13 carotenoid variables) were winsorized, capping at the 5th and 95th percentiles. Multiple comparison correction was applied to all nonparametric tests and partial correlations using the Benjamini–Hochberg false discovery rate (FDR) method. *P* values before and after FDR correction are both presented. Effect sizes (Pearson’s correlation coefficients) of 0–0.3 are interpreted as weak correlations, 0.3–0.6 as moderate correlations, and 0.6–1 as strong correlations [29].

## Results

### Descriptive summary of sample characteristics and variables of interest

The sample consisted of 51 children with 45% females and a mean age of 11.0 ± 1.9 y. Majority of participants identified as Non-Hispanic/Latino and White or Caucasian. Nearly 3 quarters of the mothers of participants had advanced degrees. Most participants came from households with annual incomes >$100,000. Over three-quarters of the participants had BMI percentile within the healthy weight range. Descriptive statistics of sample characteristics are in Table 1.

**TABLE 1:** Sample characteristics

Characteristics	*N*	Mean (SD) or *N* (%)
Age (y)	51	11.0 (1.9)
Sex, female	51	23 (45%)
Race	43	
Asian		4 (9.3%)
Black or African American		7 (16%)
White or Caucasian		29 (67%)
Other or mixed		3 (7.0%)
Ethnicity, Hispanic/Latino	42	8 (19%)
Mother education	42	
Advanced degree		33 (79%)
Bachelor’s degree		3 (7.1%)
Some college		1 (2.4%)
High school graduate		5 (12%)
Annual household income	43	
>$100,000		30 (70%)
$100,000–$70,000		4 (9.3%)
$70,000–$40,000		4 (9.3%)
≤$40,000		5 (12%)
BMI percentile	51	62.3 (26.0)
BMI percentile category		
Normal weight		39 (76%)
Overweight		3 (5.9%)
Obesity		9 (18%)

**FIGURE 1:**
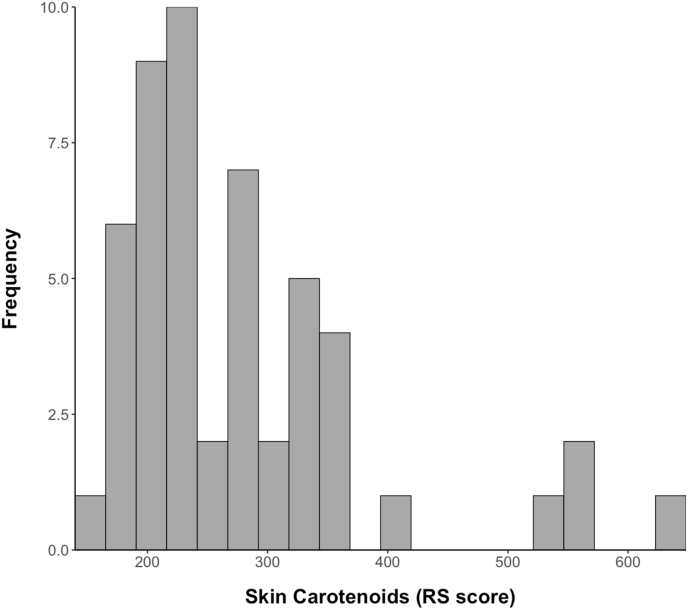
Histogram showing the distribution of RS scores (*N* = 51). RS, reflection spectroscopy.

Lycopene and β-carotene were the major carotenoids in the serum, making up 45.8% and 29.7% of the total serum carotenoids, respectively. β-cryptoxanthin was the third most abundant serum carotenoid, whereas lutein and zeaxanthin had the lowest serum concentrations. The mean skin carotenoid score was 277.9 ± 105.6 (Figure 1). The 3 skin carotenoid measurements demonstrated excellent reproducibility, with a mean coefficient of variation of 6.4% and an intraclass correlation coefficient of 0.959 (95% confidence interval: 0.94, 0.98), indicating high consistency among repeated measurements. Of the 51 participants, 43 provided diet records with a mean of 6.7 d recorded per participant. The missing diet records (*n* = 8) were due to participants not returning the records to the laboratory. Participants with missing diet records had higher BMI percentiles (74.6 compared with 60.0, *P =* 0.04). Other demographic characteristics, skin, and serum carotenoid concentrations did not differ significantly by diet record missingness. The mean daily energy intake was 1848 ± 468 kcal/d. Fat, carbohydrate, and protein intake contribute to ∼35%, 52%, and 14% of the total daily energy intake, respectively. Mirroring the serum composition, lycopene was the predominant dietary carotenoid, making up more than half of the total carotenoid intake, followed by β-carotene. Lutein and zeaxanthin were the third most abundant dietary carotenoids despite having relatively low serum concentrations. β-cryptoxanthin had the lowest mean intake. The Dietary Guidelines for Americans 2020–2025 recommend 2.5 cup equivalents of vegetables and 1.5 cup equivalents of fruits daily for children aged 9–13 y consuming an 1800-kcal diet [2]. The current sample met the recommendation for fruit intake but fell short of the vegetable intake guidelines. Dietary supplement intake was reported by 5 participants and included children's probiotics, children's multivitamin/multimineral supplements, vitamin C gummies, and iron tablets. These supplements did not contribute to dietary carotenoid intake, except for 1 participant who consumed a multivitamin chewable containing 80 μg of beta-carotene on 1 d. The descriptive statistics of serum, skin, and dietary carotenoids are in Table 2.

**TABLE 2:** Dietary intake, skin carotenoids, and serum carotenoids

Source	*N*	Variable	Mean (SD) [range]
Serum	51	Lycopene (μg/dL)	35.4 (17.0) [4.1–96.8]
		β-carotene (μg/dL)	23.0 (19.8) [2.7–94.2]
		β-cryptoxanthin (μg/dL)	11.2 (6.1) [3.2–32.0]
		Lutein (μg/DL)	6.1 (5.2) [1.8–32.4]
		Zeaxanthin (μg/dL)	1.6 (0.6) [1.0–4.5]
		Total carotenoids (μg/dL)	77.3 (38.1) [32.2–196.7]
Skin	51	Veggie Meter score	277.9 (105.6) [140–623]
Dietary	43	Total energy intake (kcal/d)	1848.4 (468.3) [912.5–3120.3]
		Total fat intake (g/d)	71.9 (18.6) [35.5–125.6]
		Total carbohydrate intake (g/d)	241.0 (68.5) [116.4–434.0]
		Total protein intake (g/d)	65.7 (21.7) [33.8–123.4]
		Total fruit intake (serving/d)	1.6 (1.5) [0–5.5]
		Total vegetable intake (serving/d)	1.6 (1.1) [0.1–3.9]
		Total fruit and vegetable intake (serving/d)	3.2 (1.9) [0.4–9.4]
		Dark green vegetable intake (serving/d)	0.1 (0.2) [0–0.6]
		Deep yellow vegetable intake (serving/d)	0.1 (0.2) [0–1.2]
		Tomato intake (serving/d)	0.3 (0.3) [0–1.7]
		Lycopene intake (μg)	3809.4 (3210.4) [0.003–13712.8]
		β-carotene intake (μg)	1917.9 (2291.6) [86.8–9593.1]
		Lutein and zeaxanthin intake (μg)	762.3 (622.3) [133.7–2963.2]
		α-carotene intake (μg)	478.7 (705.0) [1.8–3145.2]
		β-cryptoxanthin intake (μg)	75.5 (97.2) [0.2–444.8]
		Total carotenoid intake (μg)	7044.8 (4894.9) [874.9–22,226.4]

Abbreviations: g, gram; kcal, kilocalorie; μg, microgram, μg/dL, microgram per deciliter.

### Carotenoid status and dietary intake by demographic and anthropometric characteristics

Before FDR correction, total energy intake (1962 compared with 1674 kcal, *P =* 0.047) and total protein intake (71.0 compared with 57.7 g, *P =* 0.031) were higher among males. Age was negatively correlated with serum lutein (Spearman *r =* –0.31, *P =* 0.029) and positively correlated with total vegetable intake (Spearman *r =* 0.33, *P =* 0.033). BMI percentile moderately and positively correlated with total energy intake (*r =* 0.32, *P =* 0.035). However, none of these correlations remained significant after FDR correction. The only correlation that remained significant after FDR correction was between age and tomato intake (Spearman *r =* 0.42, *P =* 0.032). No significant income or race differences in serum, skin, or dietary carotenoids were detected.

### Correlations between serum, skin, and dietary carotenoids

**FIGURE 2:**
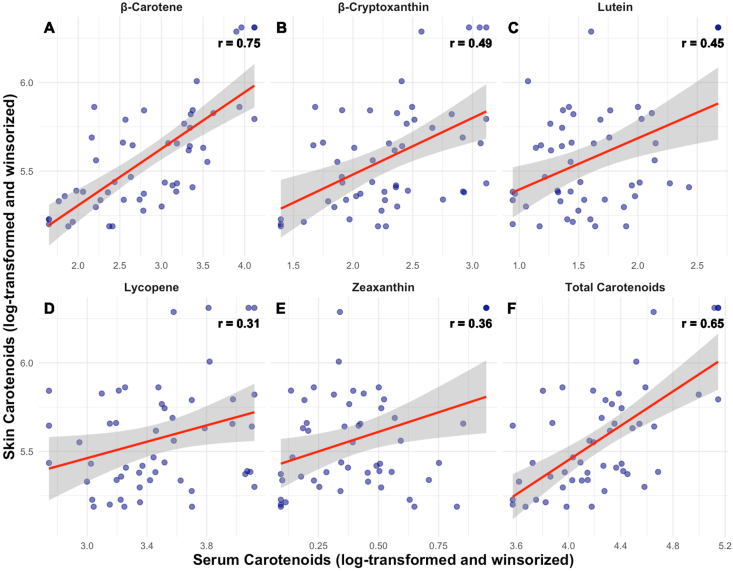
(A–F) Scatterplots of RS-assessed skin carotenoids and serum carotenoids after log transformation and winsorization (*N* = 51). RS, reflection spectroscopy.

All individual and total serum carotenoids significantly and positively correlated with skin carotenoids after log transformation and winsorization, adjusting for age, sex, and BMI percentile (Figure 2). Skin carotenoids correlated most strongly with serum β-carotene and serum total carotenoids (both *r* > 0.5). Serum β-cryptoxanthin, lutein, zeaxanthin, and lycopene concentrations showed medium effect sizes (*r =* 0.31–0.49), with the weakest correlation between skin carotenoids and serum lycopene.

Skin carotenoids moderately correlated with several dietary carotenoids (*r =* 0.37–0.48), including β-carotene, α-carotene, and lutein and zeaxanthin (as 1 variable), but did not correlate with dietary lycopene, β-cryptoxanthin, or dietary total carotenoids. Skin carotenoids also moderately correlated with dark green vegetable intake (*r =* 0.43) but did not correlate with deep yellow vegetables, tomato, total fruit, total vegetable, and total fruit and vegetable intake (Table 3).

**TABLE 3:** Partial correlations between skin, serum, and dietary carotenoids

Variable 1	Variable 2	*r*^1^	*P* values	*P* values (FDR-corrected)
*N* = 51				
Skin carotenoids	Serum lycopene	0.31^2^	0.034	0.034
	Serum β-cryptoxanthin	0.49^3^	<0.001	<0.001
	Serum β-carotene	0.75^3^	<0.001	<0.001
	Serum lutein	0.45^2^	0.001	0.002
	Serum zeaxanthin	0.36^2^	0.011	0.013
	Serum total carotenoids	0.65^3^	<0.001	<0.001
*N* = 43				
Skin carotenoids	Dietary lycopene	–0.09	0.570	0.570
	Dietary β-cryptoxanthin	0.11	0.507	0.570
	Dietary β-carotene	0.48^2^	0.002	0.0095
	Dietary α-carotene	0.38^2^	0.016	0.041
	Dietary lutein and zeaxanthin	0.37^2^	0.020	0.041
	Dietary total carotenoids	0.25	0.116	0.174
	Dark green vegetable intake	0.43^2^	0.006	0.033
	Deep yellow vegetable intake	0.35	0.027	0.080
	Tomato intake	–0.22	0.162	0.194
	Total fruit intake	0.13	0.427	0.427
	Total vegetable intake	0.29	0.067	0.134
	Total fruit and vegetable intake	0.27	0.093	0.139
Serum lycopene	Dietary β-cryptoxanthin	0.31	0.048	0.157
Serum β-cryptoxanthin	Dietary β-cryptoxanthin	0.35	0.026	0.156
	Dietary β-carotene	0.32	0.043	0.156
	Dietary lutein and zeaxanthin	0.37	0.020	0.156
Serum β-carotene	Dietary lutein and zeaxanthin	0.35	0.025	0.156
	Dietary β-carotene	0.44	0.005	0.099
	Dietary total carotenoids	0.33	0.036	0.156
Serum zeaxanthin	Dietary lutein and zeaxanthin	0.43	0.006	0.099
Serum total carotenoids	Dietary lutein and zeaxanthin	0.33	0.039	0.156
	Dietary β-carotene	0.38	0.016	0.156
	Dietary total carotenoids	0.34	0.031	0.156

Abbreviation: FDR, false discovery rate.

1 The *r* values are correlation coefficients from Pearson’s partial correlations adjusting for age, sex, and BMI percentile.

2 Indicates *P* < 0.05 after FDR-correction.

3 Indicates *P* < 0.01 after FDR-correction.

Significant correlations were observed among specific dietary and serum carotenoids without FDR correction (Table 3). Serum β-cryptoxanthin moderately correlated with dietary β-cryptoxanthin, lutein and zeaxanthin, and β-carotene (*r =* 0.32–0.37). Serum β-carotene moderately correlated with dietary β-carotene, lutein and zeaxanthin, and total carotenoids (*r =* 0.33–0.44). Serum zeaxanthin moderately correlated with dietary lutein and zeaxanthin (*r =* 0.43). Serum lycopene moderately correlated with dietary β-cryptoxanthin (*r =* 0.31). Total serum carotenoids moderately correlated with dietary lutein and zeaxanthin, β-carotene, and total dietary carotenoids (*r =* 0.33–0.38). However, none of the serum-diet carotenoid correlations remained significantly after FDR correction.

## Discussion

This study demonstrated that RS-assessed skin carotenoids significantly correlated with serum carotenoids in a sample of school-aged children, adjusting for age, sex, and BMI percentile. Specifically, skin carotenoids showed strong correlations with serum β-carotene and total carotenoids, and moderate correlations with serum β-cryptoxanthin, lutein, zeaxanthin, and lycopene, in descending order of correlation strength. All correlations were robust, remaining statistically significant after FDR correction. This study contributes to the literature by determining the correlations between individual serum carotenoids and RS-assessed skin carotenoids in a child sample, supporting the utility of RS as a noninvasive biomarker of carotenoid status in school-aged children.

The significant correlations observed in our study between RS-assessed skin carotenoids and blood concentrations of carotenoids are consistent with previous research among children using RRS. Total serum carotenoids in our sample (77.3 μg/dL) were much higher than those reported in newborns (5.72 μg/dL) and slightly higher than those in 5–17 y olds (67 μg/dL) [30,31]. Similar to our findings, previous studies have found moderate to strong correlations between total plasma or serum carotenoids and RRS-assessed skin carotenoids in children aged 0–12 y with normal weight BMI percentile (*r =* 0.39–0.78) [12,[30], [31], [32], [33]]. One study examined the correlations between RRS-assessed skin carotenoids and 2 serum carotenoid species, reporting a strong correlation with lutein (*r =* 0.52) and a moderate correlation with zeaxanthin (*r =* 0.39) in a sample of infants, toddlers, and children aged ≤7 y [33]. Our study extends the literature by providing correlations between RS-assessed skin carotenoids and individual serum carotenoids. Understanding these individual correlations has important implications for interpreting RS scores, as different carotenoids have distinct biochemical properties and preferential tissue deposition patterns [10,23]. For example, β-carotene, α-carotene, and lycopene are highly hydrophobic and preferentially accumulate in the skin, whereas lutein and zeaxanthin are more polar than carotenes and comprise majority of the carotenoids found in the macula and brain [10,34]. Individual correlations are particularly critical for RS validation, as RS measurements are less specific and precise than RRS and cannot differentiate individual carotenoids within its composite scores. Thus, determining individual correlations could help identify potential measurement bias in RS by examining whether RS detects all carotenoids equally well.

The correlations between RS-assessed skin carotenoids and total serum carotenoids in our study yielded similar effect sizes as studies in adults (*r =* 0.65–0.81), despite our child sample having lower absolute serum carotenoid concentrations compared with adult samples (129–161 μg/dL) [16,[18], [19], [20],^35^]. This suggests that RS may perform consistently across age groups. Consistent with adult studies, β-carotene showed the strongest correlation with skin carotenoids in our sample, supporting its role as a primary contributor to RS measurements [18,35,36]. However, correlations for other individual carotenoids varied more substantially between studies, particularly for lycopene. The correlation between serum lycopene and skin carotenoids in our study (*r =* 0.31) was similar to that observed in Japanese adults (*r =* 0.28) but differed markedly from that in United States adults where no significant correlation was found (*r =* 0.04–0.07) [18,35]. In our sample, lycopene was the most abundant carotenoid in both blood and diet, yet showed the weakest, albeit statistically significant, correlation with RS-assessed skin carotenoids. This suggests that RS may be less accurate for assessing lycopene intake compared with other carotenoids. Additionally, in adult feeding trials, α- and β-carotene-rich juice yielded higher increases in skin carotenoids compared with lycopene-rich juice [37,38]. Collectively, the variable correlation strengths and dose-response relationships between lycopene and skin carotenoids highlight the need for RS validation studies examining individual serum carotenoids across populations differing in age, race, ethnicity, and geography to ensure accurate clinical interpretation of RS measurements.

Although we found moderate correlations between dietary β-carotene, α-carotene, and lutein/zeaxanthin with skin carotenoids (*r =* 0.37–0.48), the corresponding serum carotenoids showed stronger associations (*r =* 0.46–0.75). Similarly, our food group analysis revealed that RS-assessed skin carotenoids correlated only with dark green vegetable intake, with no significant associations observed for tomato, total fruit, total vegetable, or combined fruit and vegetable intake. In contrast, RRS-assessed skin carotenoids were correlated with orange vegetables, leafy vegetables, total vegetables, vegetable servings, fruit servings, and total carotenoid intake in a sample of 4th and 5th graders [32]. This selective correlation pattern suggests that RS may be more sensitive to specific carotenoid sources, compared with RRS measurements. Previous studies in children have shown weak or null correlations between dietary and skin carotenoids. A study of Latino children found weak but significant correlations between 24-h recall-assessed total carotenoid intake and RRS-assessed skin carotenoids, whereas a longitudinal study of toddlers also using diet recalls detected no correlations [39,40]. Three studies using FFQ showed similarly mixed results, notably employing different questionnaires assessing intake over varying timeframes (i.e., past day, week, or month) and number of questions ranging from 5 to 38 questions [[41], [42], [43]]. Studies examining RS-assessed skin carotenoids and food groups have shown comparable heterogeneity [42,44,45]. In a sample of 264 children across multiple school levels, RS scores did not correlate with total FV liking, intake, or individual food items [44]. Another study with children of similar age to ours (9–12 y) found correlations between RS scores and total vegetable intake frequency, but not with orange and green vegetable or fruit frequencies [45]. Conversely, a study of younger children (3–5 y) showed significant correlations between RS scores and total FV and fruit frequency scores, but not vegetables [42]. Notably, the reporting source varied across studies, with children self-reporting in the first 2 studies compared with parent-reported data in the younger sample. Several factors may contribute to these variable correlations. First, correlation strength may vary across populations with different dietary patterns and demographic characteristics. For example, lycopene was the most abundant dietary carotenoid in our sample, as observed in another child sample [46]. In contrast, 2 studies among toddlers reported β-carotene as the most abundant dietary carotenoid, almost twice the amount of lycopene intake in 1 study [40,41]. Second, variations in dietary assessment methods across studies may influence correlation strengths. The half-life of skin carotenoids is estimated to be 8 wk, whereas dietary assessment tools have different timeframes, from intake over the previous day or month to general frequency questionnaires (e.g., “how many vegetables do you eat each day?”) [42,43,47,48]. Evidence from an adult study suggests that dietary carotenoids were more strongly correlated with both RSS- and RS-assessed skin carotenoids when examined over multiple time points across a year compared with single time point measurements [19]. Future studies examining the correlations between dietary and skin carotenoids may benefit from considering both the impact of dietary assessment methodology and the selective nature of RS measurements, which appear to reflect specific carotenoid sources rather than comprehensive FV intake.

We observed moderate correlations (*r =* 0.31–0.44) between several dietary and serum carotenoids; however, none remained significant after FDR correction. The lack of statistical significance likely reflects insufficient power, given the moderate strength of the correlations. Nonetheless, these correlations should be interpreted with caution and confirmed in future fully powered studies. The strongest correlation was between dietary and serum β-carotene, consistent with findings in both child and adult studies [18,31,49]. In contrast, dietary lycopene showed no correlation with serum lycopene or any other serum carotenoids, despite being the most abundant carotenoid in both dietary intake and serum in our sample. The lack of correlations between dietary and serum lycopene has been reported in both children and adults [18,31]. The correlations between dietary and serum carotenoids may depend on the dietary assessment tool used and the populations. For example, studies among females with overweight and obesity and older adults did not find correlations between dietary and serum carotenoid [50,51], whereas a study with children aged on average 10.5 y detected significant correlations only with diet recall data, not FFQ [31]. Measurement errors associated with self-reported dietary assessments may further contribute to the inconsistent findings. FFQ has been reported to underestimate total energy intake and carotenoid intake, with lutein and lycopene showing the lowest validity coefficients compared with other carotenoids [52,53]. Although food records, as used in the present study, are advantageous compared with FFQs because they do not rely on memory, they have limitations, including high respondent burden and potential decreasing validity as recording days increase [54]. Additionally, underreporting of energy intake has been documented with parent-assisted food records [54], and parental reporting may be subject to portion size estimation errors and social desirability bias [8], which could attenuate dietary–serum correlations and contribute to loss of statistical significance after multiple comparison correction. Lastly, the bioavailability of carotenoids varies substantially depending on food matrix and preparation methods, which could affect their blood concentrations. For example, the bioavailability of β-carotene varies between 5% and 65% [23].

The strengths and limitations of the present study offer several future directions. To our knowledge, this was the first study to validate RS in children against individual and total serum carotenoids analyzed by HPLC. We were also able to include individual dietary carotenoid data from detailed food records over 7 d. However, this method remains susceptible to parental reporting bias and measurement errors [8]. Another notable strength was the inclusion of FDR correction for multiple comparisons, which increases the confidence in the correlations detected in our study between serum and skin carotenoids. One limitation was that our sample predominantly consisted of high-income families with an annual household income over $100,000. Although RS-assessed skin carotenoids did not differ by income categories in the present study, previous research has shown significantly lower skin carotenoids among children from families receiving federal food assistance and from low-income schools [39,45]. Children from households earning <130% of the United States federal poverty level are significantly less likely to have consumed any dark green vegetables on a given day compared with those from families earning >350% poverty level [55]. However, some studies suggest that children’s diet quality may be less affected by household economic hardships compared with adults [56,57]. Our sample was also demographically homogeneous, consisting predominantly of children identifying as non-Hispanic Caucasian with normal weight BMI percentiles. Although the Veggie Meter scoring algorithm corrects for melanin and other chromophore interference [16], and 1 diverse child sample (*n* = 143, 43% Hispanic, 37% Caucasian, 18% Other) found no racial or ethnic differences in RS-assessed skin carotenoids [45], RS validation research across racially diverse pediatric samples remains sparse. Additionally, higher BMI has been associated with lower skin carotenoids [28], yet our sample had limited representation of children with overweight and obesity. Given these demographic characteristics and our relatively small sample size, future validation studies could benefit from recruiting larger, more diverse samples that include greater variability in household income, racial and ethnic backgrounds, and weight status to establish the broader generalizability and clinical utility of RS in children. Additionally, the cross-sectional design limited our ability to examine the potential seasonal effect and intravariability on skin, serum, and dietary carotenoids. Jahn et al. [19] found varying correlation strengths between serum, skin, and dietary carotenoids when comparing cross-sectional compared with longitudinal data over 1 y in adult females. Thus, future studies could utilize a longitudinal design to further explore the long-term reliability of RS and its correlations with serum and dietary measures.

In conclusion, this study demonstrated significant correlations between RS-assessed skin carotenoids and serum carotenoids, supporting the utility of RS as an objective measurement of carotenoid status among school-aged children. However, correlation strengths varied considerably by carotenoid species, with lycopene demonstrating notably weaker correlations compared with β-carotene and other carotenoids. Additionally, RS scores showed stronger correlations with individual dietary carotenoids than with FV food groups, correlating only with dark green vegetable intake among those examined. Our findings extend existing literature by establishing differential correlation strengths across individual serum carotenoids, which may reflect distinct tissue distribution patterns and enable more accurate interpretation of RS scores. These results provide important evidence for RS application in school-aged children, while highlighting the need for longitudinal validation studies across diverse groups.

## Author contributions

The authors’ responsibilities were as follows – RL, NAK: designed research; LMR, MB, JWE: conducted research; RL: analyzed data; RL, NAK: wrote the paper; NAK: had primary responsibility for final content; and all authors: read and approved the final manuscript.

## Data availability

Data described in the manuscript, code book, and analytic code will be made available on request pending application and approval.

## Funding

This work was funded by the Egg Nutrition Center and the Division of Nutritional Sciences, University of Illinois Urbana-Champaign. The funders had no role in the design, implementation, analysis, and interpretation of the data.

## Conflict of interest

NK reports financial support was provided by Egg Nutrition Center. If there are other authors, they declare that they have no known competing financial interests or personal relationships that could have appeared to influence the work reported in this paper.
